# Topics of Public Interest

**Published:** 1916-11

**Authors:** 


### TOPICS OF PUBLIC INTEREST

---

**Rural Sanitation.** The U. S. Public Health Service made surveys in 1914 showing that 1% of rural homes had sanitary toilets and that over 50% used contaminated water. In 1916, the same service is asking: Do you know that a sanitary water-closet is better than a privy? and: Do you know that it is a bad thing to drink polluted water? Or, if not, it is distributing the same kind of questions and most medical journals seem to fall for printing them.

---

**Narcotic Law Decisions.** The federal court, N. D., N. Y. (U. S. vs. Curtis, 229 Fed. 288) has ruled that both the prescriber and the dispenser of a prescription for an unusually and unreasonably large amount of a narcotic drug are guilty of an offense, unless the prescription indicates the reason and necessity for demanding such an amount. The federal court S. D., Ohio, Tucker vs. Williamson, holds that though a physician may under proper circumstances, distribute or dispense narcotic drugs to his patients, even one with a medical degree, may not be registered and allowed to send such drugs through the mail when most of his patients are not seen personally and when he bases most of his prescriptions on written statements of patients and prescribes for all of them alike.

---

**Mailing Poisonous Drugs.** It is probably not generally known that the U. S. Criminal Code, Section 217 absolutely prohibits the sending of poisonous drugs, as well as poisonous insects and reptiles, explosives, dangerous chemicals, etc., through the mail. Probably this law has frequently been violated by physicians, scientists, collectors, etc., without malicious intent. Indeed, as most active drugs are poisonous in the quantities often prescribed and dispensed at once, probably a great many physicians have rendered themselves technically liable to prosecution, by mailing packages of tablets to patients. The Kern-Doremus bill (Senate 6834, House 17396) recently introduced, modifies this law somewhat as to allow poisonous drugs to be shipped if properly packed and authorizes the postmaster general to make the necessary regulations. We would therefore urge our readers not to send medicines that could be considered poisonous through the mails until this amendment becomes operative and to inquire at the post office as to the regulations for their packing. As we understand the further reading of the amendment, the mailing of poisonous animals, insects and reptiles, explosives and inflammable materials—including such as may explode or ignite though not intended for such purposes—is still prohibited absolutely. As physicians are often interested in scientific hobbies and as the art of medicine depends to some degree upon such animals and substances, this prohibition should be carefully noted. It is still more important to remember that disease germs, scabs, and apparently all alcoholic liquors, as well as dangerous substances of all kinds, are unmailable. Sealing and payment of first-class postage does not exempt. DOCTOR: DO NOT FAIL TO READ THIS ITEM CAREFULLY.

---

**Bates Bill to Apply to Control of Poliomyelitis.**. The following bill is now in effect in N. Y. State. Physicians should note that it is an emergency measure, inevitably subject to contradictory interpretations and that, as no one seems to know exactly how poliomyelitis is transmitted, health officials may make demands which would not occur to the individual physician, however careful and conscientious. We therefore suggest that physicians in any way connected with a case that may, by any possibility be diagnosed as poliomyelitis, inquire of the local health officer as to exactly what is required of them.

"Section 1. Boards of health in cities and towns, or boards of selectmen in towns, may make such rules and regulations as are necessary and proper to check the spread of the disease known as infantile paralysis, and to cause its eradication by quarantine or otherwise.

"Section 2. The state department of health shall have the power to revoke or revise any such rule or regulation when they are unnecessary or unreasonable, and all such rules or regulations shall not have force or effect after Jan. 15, 1917."

---

**An Educational Lunch Room.** The Public Health Dept. of N. Y. City maintains a lunch room for employes. Recently menus have been printed with price, weight of service, calories and grams of proteid. Some such plan as this might well be placed in operation in every city, somewhere. A few years ago, there was a general disposition on the part of dieteticians to teach, and of the medical profession to learn, quantitative dietetics by some simple rule. It is scarcely necessary to point out that there is no royal road to learning food dosage, any more than drug dosage. It is only by repeated, concrete examples that a working knowledge of dietetics can be learned. Under existing conditions, a practical knowledge of dietetics by the laity is of great potential economic value. For example, most fresh vegetables aside from seeds are considered as cheap and nutritious. On the contrary, they contain very little nutriment, as retailed they are often quite expensive in proportion to either their nutriment or aesthetic value. No particular one is necessary and, indeed, the whole group may be dispensed with. For a considerable period of the past summer, potatoes have been high but poor families have bought them under the belief that they were necessary. Weight for weight, allowing for skins, potatoes have about 1/6 the caloric value of wheat flour and about 1/9 the proteid value. Until introduced from America, about 300 years ago, they were unknown as an article of food except to the Indians. The price of potatoes even with flour at war prices, has been ¾ of the latter and has probably averaged ½ for the whole summer. They do not even taste better than flour and rice, etc., similarly served as a vegetable. If there had been a popular realization that, in proportion to what was actually got in the way of nourishment, the current prices of potatoes had ranged about 3 times what they were worth, not only would there have been an enormous aggregate saving to consumers but the diminution of demand would automatically have reduced the price of potatoes to normal. We would be pleased to co-operate in a plan to establish educational lunch rooms—or rather to add the educational factor to existing lunch rooms—under proper auspices in this part of the state.

---

**Bacteria in Commercial Bottled Waters.** U. S. Dept, of Agriculture, Bulletin No. 369. Of 110 domestic springs, 47 were free from colon bacilli, 63 contained colon bacilli in samples of 10 c.c. or less. 59 of the 63 contained colon bacilli in samples of 1 c.c. which, by ordinary standards of health authorities renders them suspicious in regard to typhoid. Positive findings in amounts of 0.1 c.c., .01 and .001 occurred in 49, 31 and 10 waters, respectively, each larger group including, of course, the smaller consecutive groups.

69 of the waters gave less than 100 total bacteria per c.c. after incubation at 37° for 2 days while 14 gave counts always greater than 1000 on 6 or more individual bottles. The highest count was 191,238. The results for 57 foreign waters were slightly better but two imported waters labeled "bacteriologically pure" gave averages of 1,250 and 482 total bacteria per c.c., respectively. 4 of 6 bottles of one showed colon bacilli in 10 c.c. quantities and all six of the second gave the same result. 4 of one and 5 of the other showed colon bacilli in amounts of 1 c.c. or less.

This is a most important subject. Thousands of people who can not speak of the Standard Oil Co. calmly, pay more for drinking water than they do for kerosene, some even more than they do for gasoline. They do not stop to consider that a highly popular spring, whose water is sold by thousands of gallons daily in various cities, must be either a subterranean river of too great bulk to allow either guarding of water shed or natural filtration, or else a purely fictitious trade name. Nor do they free themselves sufficiently from poetic associations with the word spring, to act on their own experience that the average spring, not rigorously cleaned and guarded, is usually a nasty source of drinking water. This journal has consistently followed the rule to accept no advertisements of potable waters which are not safe-guarded by reliable bacteriologic reports. Meantime, let us not forget the very practical point that the average city in our territory and even the average village that has established corporation water works, has, even without sand filtration, boiling or other methods of treatment, water that is on the whole vastly safer as well as very much cheaper than the average bottled table water, if the U. S. authorities can be relied upon.

The bacteria identified in the flora of bottled waters were: B. coli, B. mycoides, B. paratyphosus, strain B, B. aerogenes, B. aurantiacus, M. citreus, B. maratimum, B. ovale, B. prodigiosus, B. fluorescens liquifaciens, B. fl. non-liq., B. subtilis, and long-chain streptococci. Molds of the genera trichoderma, penicillium, cladosporum, citromyces, fusarium, actinomyces and sporotrichum were also isolated. Some are more or less pathogenic.

---

**War Losses Among British Surgeons.** The British Medical Journal states that over 400 have been killed or died in active service, at the front, in the year ending Aug. 1916.

**The Cocaine Habit** has been found to be quite common among British soldiers and drastic regulations have been made to check it.

---

**Physical Condition of Volunteers.** In mustering state guards into the U. S. service, the following percentages of rejections for physical reasons, have been made: Iowa, 8, Ill. 10, Mo. 12, Minn. 13, Mich. and Neb. 15, Kas. 16, Wis. 17, Ind. 21, Ohio over 30.

---

**Centenarian.** Samuel McKee, born in Ireland, Nov. 2, 1813, of Scotch ancestry, died in Tidioute, Pa., Oct. 3, one month short of 102 years. He had had 18 children.

---

**Automobile Caution.** Do not forget that the late fall is the time when most radiators burst from freezing. It is not the average but the minimum night temperature that counts. 10% of denatured alcohol will protect to about 25° F. and is usually sufficient in this climate till early in December. Remember, too that this is the season when it is most important to have your starter working and when it is most likely to get out of order. Lighting imposes more strain on the battery in the short days and there is less high-speed running to charge the battery. A weak battery may freeze. Have your battery inspected, if necessary overhauled and expect to have to get it recharged from a station once or twice during the winter. Some automobilists fail to realize that a starter is merely a substitute for the strong arm in spinning a motor that does not start easily under any conditions. In most starting and lighting equipments, the motor is cranked on the magneto, which is not quite so quick as a battery. The starter, like a strong mechanic, may be able to get the engine running under unfavorable conditions but, to secure good results, it is just as necessary to have the spark plugs clean and properly adjusted, to have all wiring connections bright and tight, to enrich the mixture by choking or priming and by setting the carburetor adjustment for cold weather, as in a hand cranked car.

---

**Niagara Co. Tuberculosis Hospital.** The supervisors have employed Chester R. Phelps of Niagara Falls as architect. He has made plans for four other county buildings. The committee, after an investigation tour of the state decided that the Poughkeepsie and Rochester hospitals were best adapted as models. An additional strip of land has been bought to afford an entrance to the site for the hospital, near Lockport.

**The Plattsburg Military Training Camp** closed October 12. 12,000 men received training in five periods, this summer.

---

**Army Medical Corps.** Examinations will be held early in January for appointment of first lieutenants. There are 228 vacancies. Examinations will be held at various stations, to suit the convenience of candidates. Application should be made to the Surgeon General, U. S. Army, Washington, D. C.

---

**Popular Lectures on Medical Topics.** The B. Merrill Ricketts Experimental Surgical Research Laboratory of Cincinnati, will give a series of lectures on Saturday evenings, in Cincinnati. This precedent may well be followed by other cities. It may be pointed out that practically all friction in regard to such courses is avoided by the simple expedient of eliminating local talent.

---

**The Balkan Relief Fund** has been established with Rev. Frederick Lynch, Editor of The Christian Work, 70 Fifth Ave., N. Y., as treasurer. A ship will soon sail, loaded with supplies. It is pointed out that, while Belgians have been well fed, 200,000 Albanians have died of starvation on account of the present war. Wm. W. Howard, lately returned from his third trip to the war zone, reports corn at $50 a bushel, flour at $70 a sack, macaroni at $5 a pound. The distress is great and there should be no likes and dislikes in our charity.

---

**Professional Pride.** A writer in an exchange tells of a call from a Radiographer, whom he found a most interesting guest and treated as a distinguished colleague, until in the course of the conversation, it developed that he was merely a photographic expert and not a medical graduate, when his entertainer had no further use for him. It strikes us that this is carrying professional esprit de corps too far. Shall we adopt the same course with chemists and bacteriologists with Ph. D. degrees, which are a good deal harder to get than M. D.'s. Of course, if the guest had deliberately misrepresented himself in any way, even implicitly by claiming for a mere assistant the courtesies due a worker of standing, the case would have been different but this does not seem to have been the case. Neither medical nor premedical courses could afford a basis for roentgenology, except for workers of recent date and except in regard to certain points in anatomy and physiology which, it must be confessed, do not apply very directly or even accurately to roentgenologic study. The roentgenologist with a medical degree has the legal right to supplement his examinations with medical or surgical therapeutics but he seldom does so in any direct sense. As a matter of fact, roentgenology, with or without a medical degree, is largely acquired by informal study of a personal nature. It is one thing to regard askance the man who engages in the practice of medicine without formal training and license, quite another to place in the same category the earnest workers along lines of science utilized by medicine, and who feel that the limitation of their work does not demand the securing of a medical degree.

**Advance in Cost for City Patients.** The Buffalo councilmen have been petitioned by the hospitals for an increase in rates from $7 to $9.80. Dr. R. R. Ross of the General Hospital presented statistics showing that the actual cost now ranges from $12.46 to $14.10, in the independent hospitals, and is over $12 in the Municipal Hospital and over $13 at the J. N. Adam Memorial Hospital.

**Chautauqua Co. Tuberculosis Hospital Site.** By will of the late Mrs. Elizabeth M. Newton of Fredonia, $150,000 was left for the establishment of a hospital and a site of 180 acres was favorably considered by the trustees. This site is the Pierson farm on upper Cassadaga Lake, the price being $19,999. At a hearing before the Deputy State Commissioner of Health, Dr. L. R. Williams of Albany, and the Fredonia town Health Officer, Dr. V. M. Griswold, Oct. 13, opposition to the site was made by representatives of the Lily Dale Assembly Assn. It may be stated that the level of this lake is 1305 feet above sea level, the ground rising rapidly along its shores to 1400 and even 1500 ft. The lake is about ½ mile wide and not quite a mile long, connecting with two other lakes so that the combined lake and swamp area is about ¾ mile in width and not quite 2 miles in length, with a projection of flat land about ¾ mile square, into the eastern side of this lake area, on which is located the Lily Dale summer colony. These lakes drain into the Sassadag Creek, and ultimately into the Allegany and Ohio Rivers, the grade for the first 30 or 40 miles being on the average about 1 ft. to the mile. It will be noted that, except for the swampy ground in the vicinity and the chances of pollution of the lake from its steep but not very extensive water-shed, the site is almost ideal from the standpoint of its prospective inmates. Whether, from the standpoint of its neighbors, any appreciable danger exists, should be impartially determined, remembering that the lake lacks, on the one hand, the volume and extent favoring self-purification and, on the other hand, the rapid change of water noted in many small lakes. It should be borne in mind that in the establishment of County Tuberculosis Hospitals, we are building for 50 years ahead. We personally hold the opinion that if tuberculosis can be universally and efficiently segregated, the disease can be practically stamped out in this time. Thus, even from the humanitarian standpoint in the long run, the question of whether a site where tuberculous patients are assembled is a danger to the neighborhood is more important than whether it is well adapted to the care of its prospective inmates. The fact that the conveyance of tubercle vacilli cannot usually be traced to a definite source, increases rather than diminishes the need of caution in this respect. It is still subjudice whether there is such a thing as closed tuberculosis from the sanitary standpoint. The theory of elimination of bacilli by the faeces is not absolutely demonstrated but has recently received substantial support. We would not go so far as to express the opinion that a tuberculosis hospital, especially for incipient cases, located on Cassadaga Lake, would endanger the permanent and summer residents of the vicinity but a good many persons would undoubtedly prefer to take no chances and we frankly admit being in this "safety first" group.

---

**After-Care for Paralysed Children.** The State Dept. of Health is formulating a plan to provide prosthetic apparatus, professional supervision and even transportation in needy cases. Up to Oct. 1, 3,301 cases of poliomyelitis have occurred in the state outside of N. Y. City. 589 have died, most of the remainder will have some loss of muscular power.

---

**Christian Science Treatment Legal.** The Court of Appeals, at Albany, in passing upon a case originally brought up by the Medical Society of the Co. of N. Y. in 1911, reverses the decision of a lower court which imposed a fine of $100 upon a healer, remits the fine and orders a new trial. The opinion expressed is that practicing medicine is not in itself a crime at common law and that the statute requiring license of practitioners is itself explicitly qualified by the statement that it shall not be construed to affect the practice of the tenets of any church. However, it does not seem to us that this verdict can be heralded as a great triumph over the medical profession since the opinion proceeds:

"We think the exception in the statute in this state is broad enough to permit offering prayer for the healing of disease in accordance with the recognized tenets of the Christian Science church. . . .

"A person should not be allowed to assume to practice the tenets of the Christian Science or any other church as a shield to cover a business undertaking. When a person claims to be practicing the religious tenets of any church, particularly where compensation is taken therefor and the practice is apart from a church edifice or the sanctity of the home of the applicant, the question whether such person is within the exception should be left to a jury as a question of fact.''

Much as we may feel that the sick and injured should be assured of the best and most highly trained assistance, especially when, by reason of infancy or any other cause, they are incapable of forming mature opinions of their own, we can scarcely object to the exercise of the right of a personal choice. As we understand the animus of the original suit, it was not to establish the privilege of the medical profession to force its attendance on those unwilling to receive it.

---

**Poliomyelitis.** There is a rather vague preliminary report that necropsies at Johns Hopkins have shown characteristic intestinal lesions. This indicates that the disease must be viewed from a different standpoint but does not, thus far, explain the peculiar inconsistencies of its transmission.

---

**Increase in Price of Milk.** Grade A pasteurized milk 10 cents; grade B, 9 cents; Larkin Jersey raw milk and Pine Grove Farm certified, 18 cents a quart, announced by Q. C. Dairy Co., Buffalo, effective Oct. 23. In this connection it may be remarked that the State Health Dept. has recently argued against compulsory pasteurization as tending to limit the milk supply to large dealers and as acting mainly to prevent souring.

---

**The N. Y. Skin and Cancer Hospital** announces a course of clinical lectures on diseases of the skin by Dr. L. Duncan Bulkley, assisted by the attending staff. Lectures will be held in the Out-Patient Hall of the Hospital, Wednesday afternoons, at 4:15 o'clock, beginning Nov. 1. The medical profession will be admitted on presentation of their cards.

---

**Hospital for Springville.** The will of Bertrand Chaffee, who died Oct. 3, provides that, after the death of his widow, the family home shall pass to the village as the site of the Bertrand Chaffee Memorial Hospital.

---

**Poliomyelitis Epidemic.** The Literary Digest publishes at the front of its issue of Oct. 14, a chart showing the curve of incidence and mortality for N. Y. City. From June 5 to 20, the case reports varied from 0 to 2 or 3 a day. Till July 11, the curve rose sharply to 195 and then fell to 80 on July 24.

It reached its maximum with 217 cases Aug. 3 and then fell gradually to 10 cases Oct. 2. The death curve is more regular, reached the maximum of 57 Aug. 10 and, since Sept. 8, has been 10 or a few less a day.

---

**Poliomyelitis Case in Albion.** The first case in Orleans Co. developed Oct. 16, in a boy aged 6, attending the Central Public School which has been ordered closed for 2 weeks.

---

**National Licenses for Medical Practice.**. 10 candidates took the examination of the voluntary national board previously described, on Oct. 16.

---

**Agricultural Use of Gas Helmets.** Threshers in Lancaster Co., Pa., have adopted a respirator helmet similar to those used in the war zone, to protect from dust.

---

**Gasoline Tanks.** Automatic pumps have been found by investigation of the federal authorities to give short measure in about 80% of cases.

---

**Standard Names for Automobile Parts** have been adopted by the American Society of Automobile Engineers. 19 divisions have been recognized with 70 subdivisions, the total technical vocabulary being about 700 terms. This fact should be remembered by those who claim that the average mechanic knows only a thousand words in all.

---

A recent investigation made by the U. S. Public Health Service in connection with studies of rural school children showed that 49.3 per cent had defective teeth, 21.1 per cent had two or more missing teeth, and only 16.9 per cent had had dental attention. Over 14 per cent never used a tooth brush, 58.2 per cent used one occasionally and only 27.4 per cent used one daily.

---

**The Trend Toward Decimal Systems.** We have just received an English exchange bearing a postage stamp marked "Three Half Pence." This is a straw which shows which way the wind blows. It has already been formally suggested in England that the dollar be adopted as the monetary unit, as it is already familiar both in financial circles and popularly. "I'll bet you a dollar" has long been current in England. In most of the countries of Europe south of Holland, Germany and Austria, the franc (or its equivalent passing current in all countries of the corresponding league) is practically subdivided, not into hundredths but into twentieths, practically equal to our cent. The German mark is, at present, of almost exactly the same value as the franc so that the 5 pfennig piece is also practically a cent. Luxemburg, which has for many years legalized both German and French coinage and has none of its own gets around the difficulty by thinking in American cents—of course, without realizing the fact—and making change between the two coinages just as we would do, thinking of the mark as a quarter and the franc as a twenty-cent piece. Most of the other countries of Europe have decimal monetary systems easily convertible into dollars and cents.

Meantime, the war has familiarized England and its colonies with the metric system and has clearly shown the advantages even in military preparedness due to its use. England and the U. S. are the only civilized countries of any importance that have not already long used this system and it is almost universally employed in all sciences, even applied, in the U. S., up to the point of commercial dealings. A little progressiveness and co-operative spirit at the close of the war ought to bring about international unity in the adoption of decimal systems and, aside from the loss of human life and antiquities, such a standardization of measures would probably result in increased efficiency which would balance the material losses of the war.

---

**Milk Code for Buffalo.** A hearing was held Oct. 17 and another will be held Nov. 3, or held subject to further consideration. It has been provisionally agreed, to meet the demands of the milkmen that bacterial counts shall not be published; that pasteurization shall not be compulsory; that skim milk shall not be labeled; and that ice shall not be required on wagons making night deliveries. It was stated that some housewives considered a low bacterial count as indicating that they were not receiving their money's worth.

---

**Milk Prices in Dunkirk.** The Dunkirk Milk Producers' Assn., supplying 2500-3000 quarts a day out of a total of 4.000-4,200, has increased the price paid to producers from 16.5-18.5 cents a gallon to a flat rate of 5 cents a quart for May, June and July, when it will be 4.5. Retail prices are 9 cents a quart, 5 cents a pint. (Note: A considerable factor in the difference between wholesale and retail prices is the expense of bottles which have to be renewed about once a month).

---

**Civil Service Commission.** Examinations for the State, County and Village Service, Dec. 2, 1916. Application blanks will not be sent out by mail after Nov. 20. Application blanks received at the office of the Commission after Nov. 22, will not be accepted.

188. Woman Physician, (Regular or Homeopathic) State Hospitals and Institutions. $1000 to $1500 and maintenance. Candidates must be licensed medical practitioners of the State of New York, and must have had at least one year's experience on the medical staff of a hospital or three years' experience in the general practice of medicine. Subjects of examination and relative weights: Anatomy, physiology, materia medica, therapeutics, chemistry, obstetrics, surgery, theory and practice, 7; experience, education and personal qualifications, 3. Candidates may be summoned for an interview with the examiners. Open to non-residents.

211. Physician, State Prisons and Reformatories. Salary $2000 without maintenance. Examination open only to men who are licensed medical practitioners in this State, who are not less than 25 years of age and who have had at least three years' practice. Subjects of examination and relative weights: Written examination, covering anatomy, physiology, chemistry, materia medica, therapeutics, surgery, theory and practice, 4; laboratory work, prison sanitation and hygiene, 2; education, experience and personal qualifications, 4. Candidates who attain at least 60 per cent in the written examination may be summoned before the examining committee in connection with the rating of the last mentioned subject. It is expected that one appointment will be made at Sing Sing Prison in the near future.

212. Deputy Medical Examiner, Bureau of Deportation, State Hospital Commission. $3500. Applicants must be physicians licensed to practice in New York State, and with not less than five years' experience in the practice of medicine. Minimum age limit 30 years. This position was established in accordance with the provisions of Chapter 121, Laws of 1912, to which reference should be made for a description of the functions of the Bureau of Deportation. Applicants will be expected to have a knowledge of the Insanity Law, and experience in the care and treatment of the committed or alleged insane in the New York State Hospitals or elsewhere, or knowledge and experience of the problem of the alien insane, and their deportation. Subject of examination and relative weights: Questions relative to the duties of the position as outlined above, 4; experience and personal qualifications, 6. Candidates may be summoned for an interview with the examiners.

213. Nurse, State Commission for the Blind. Open to men and women. Salary $1200. Candidates must be graduates of a nurse training school registered by the State Education Department. This examination is intended to provide a list of eligibles for the position of eye nurse, and candidates must have had special training or experience as such. No written examination will be given but candidates will be rated on their age, education, preliminary training and experience as determined from the sworn statements in their applications and by inquiries which the Commission may direct to proper sources among previous employers, etc. An oral interview may also be given. Open to non-residents and non-citizens.

214. Trained Nurse, State Institutions. Open to men and women, $420 to $600 and maintenance. Candidates must be graduates of a nurse training school registered by the State Education Department. This examination is intended to provide a list of eligibles for the positions of trained nurse, chief nurse and hospital matron in the various State institutions, excepting State hospitals for the insane. No written examination will be given but candidates will be rated on their age, education, preliminary training, and experience, as determined from the sworn statements in their applications and by inquiries which the Commission may direct to proper sources among previous employers, etc. Candidates may be summoned for an interview with the examiners. Open to non-residents and non-citizens.

215. Matron, (Reception House), State Reformatory Institutions for Females. $720 to $840 and maintenance. Subjects of examination and relative weights: Letter-writing, 1; arithmetic (fundamental operations, and United States money), 1; questions relating to the duties of the position, 3; experience, education and personal qualifications, 5. Candidates should in their applications, submit a careful review of their past experience, with special emphasis on matters tending to specially qualify for this position, the duties of which are such as to require a woman of strong physique and personality and a good disciplinarian. Candidates may be summoned for an interview with the examiners. An applicant who is at present, or has been previously, employed at one of the institutions for females at Albion, Bedford or Hudson must file with her application a letter of recommendation from the present Superintendent of the Institution where last employed. One appointment is expected at the Institution at Bedford Hills.

216. Matron, (Cottage), State Reformatory Institutions for Females. $480 to $600 and maintenance. Subjects of examination and relative weights: Letter-writing, 1; arithmetic (fundamental operations, and United States money), 1; questions relating to the duties of the position, 3; experience, education and personal qualifications, 5. Candidates should in their applications, submit a careful review of their past experience, with special emphasis on matters tending to qualify for this position. An applicant who is at present, or has been previously employed at one of the institutions for females at Albion, Bedford or Hudson must file with her application a letter of recommendation from the present Superintendent of the Institution where last employed.

223. Dentist, Monroe County Service. Open to men only. Candidates must be graduates of a recognized dental college, and eligible to enter the State licensing examination. They will not be required to appear for written examination but they will be rated on education, experience and personal qualifications as set forth in their formal applications,, and as determined from inquiries which the Commission may direct to proper sources of information. A vacancy exists at the Iola Sanatorium at $60 per month.

---

**Poliomyelitis.** For the week ending Oct. 21, the number of cases in N. Y. State, exclusive of N. Y. City, was 53, as compared with 86 for the week ending Oct. 14 and 127 for the week ending Oct. 7.

